# Artificial bionic taste sensors coupled with chemometrics for rapid detection of beef adulteration

**DOI:** 10.1002/fsn3.2494

**Published:** 2021-07-29

**Authors:** Biao Lu, Fangkai Han, Joshua H. Aheto, Marwan M. A. Rashed, Zhenggao Pan

**Affiliations:** ^1^ School of Information and Engineering Suzhou University Suzhou China; ^2^ School of Biological and Food Engineering Suzhou University Suzhou China; ^3^ School of Food and Biological Engineering Jiangsu University Zhenjiang China

**Keywords:** beef adulteration, electronic tongue, extreme learn machine, rapid determination

## Abstract

The purpose of this study was to investigate the potential of taste sensors coupled with chemometrics for rapid determination of beef adulteration. A total of 228 minced meat samples were prepared and analyzed via raw ground beef mixed separately with chicken, duck, and pork in the range of 0 ~ 50% by weight at 10% intervals. Total sugars, protein, fat, and ash contents were also measured to validate the differences between raw meats. For sensing the water‐soluble chemicals in the meats, an electronic tongue based on multifrequency large‐amplitude pulses and six metal electrodes (platinum, gold, palladium, tungsten, titanium, and silver) was employed. Fisher linear discriminant analysis (Fisher LDA) and extreme learning machine (ELM) were used to model the identification of raw and the adulterated meats. While an adulterant was detected, the level of adulteration was predicted using partial least squares (PLS) and ELM and the results compared. The results showed that superior recognition models derived from ELM were obtained, as the recognition rates for the independent samples in different meat groups were all over 90%; ELM models were more precisely than PLS models for prediction of the adulteration levels of beef mixed with chicken, duck, and pork, with root mean squares error for the independent samples of 0.33, 0.18, and 0.38% and coefficients of variance of 0.914, 0.956, and 0.928, respectively. The results suggested that taste sensors combined with ELM could be useful in the rapid detection of beef adulterated with other meats.

## Graphical abstract

E‐tongue for rapid determination of beef adulteration.

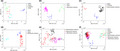

## 1 INTRODUCTION

Meat and meat products are popular food commodities around the world due to their high nutritional value and unique flavor. The high demand for meat and meat products makes them an appealing target for adulteration by dishonest traders for financial gains. Meat adulteration has a long history and is still a serious problem around the world, despite the fact that it is prohibited by various national and international laws. For several years, beef has been targeted for adulteration with low‐price meats such as chicken, duck and pork in order to increase financial return, particularly minced beef products (Shi et al., 2019). Such beef adulteration not only constitutes consumer fraud and commercial malpractice, but it also raises concerns for people who are allergic to specific meat species or have religious or ethical aversions (Kamruzzaman et al., 2015). To prevent such meat adulteration, rapid and reliable authentication techniques are required.

One of the key challenges confronting food regulatory authorities in confronting meat adulteration is directly related with meat processing techniques. For instance, the development of advanced techniques for food processing such as deboning, mincing, chopping, emulsification, and other preprocessing procedures have made the identification of meat species become more difficult due to the changes in external morphological features (Shi et al., 2019). As a result, conventional sensory evaluation and morphological discrimination should be replaced by more accurate and sensitive analytical techniques. Currently, various adulterant analytical techniques have been reported, as comprehensively reviewed in these articles (Alikord et al., 2018; Böhme et al., 2019; He et al., 2020; Li et al., 2020; Zia et al., 2020). The most specific and sensitive techniques presented are DNA‐and protein‐based methods such as polymerase chain reaction (PCR) and enzyme‐linked immunosorbent assay (ELISA) (Boyacı et al., 2014). These methods are reliable, but they are time‐consuming and require sophisticated laboratory analyses (Zhao et al., 2019). As a result, an analytical technique offering rapid result acquisition and convenience must be proposed (Rohman et al., 2011).

To that end, rapid spectroscopy methods such as near infrared (Alamprese et al., 2016; Ding & Xu, 2000; Leng et al., 2020; Rohman et al., 2011), mid‐infrared (Al‐Jowder et al., 2002), Raman spectroscopy (Boyacı et al., 2014), multispectral image (Ropodi et al., 2017; Ropodi et al., 2015), hyperspectral (Chen et al., 2018; Jiang et al., 2019; Kamruzzaman et al., 2016; Kamruzzaman et al., 2015; Zhao et al., 2019), and combination of UV‐visible, near infrared, and mid‐infrared spectroscopy (Alamprese et al., 2013) have been utilized for determination of beef adulterated with other meats. However, for homogeneous biological tissues, NIRS using continuous wave can be used to detect the signal of the deep position is only approximate 14 mm (CAO et al., 2006). The limitation of measuring depth may be the most significant barrier to the practical application of spectral techniques to detect meat adulteration. Electronic tongue (E‐tongue) is an appealing option because it is quick, simple, and above all environmentally friendly.

Since its inception, E‐tongue has been widely used for food quality analysis as an imitation of taste perception and sensory evaluation. E‐tongue, on the other hand is equipped with high cross sensitive and nonspecific sensors that detect taste compounds/dissolved components, as opposed to spectroscopy technology. Some notable applications of E‐tongue include the prediction of sodium chloride, nitrate, nitrite, and anions nitrate contents in minced meat of pork (Campos et al., 2010; Labrador et al., 2010); monitoring of physical–chemical and microbiological changes in fresh pork meat (Gil et al., 2011); assessment of red meat origins (beef, goat, and sheep) and their storage time (Haddi et al., 2015); identification and prediction of the chemical compositions of beef from different breed cattle (Xinzhuang et al., 2015); sensing taste attributes of dry‐ and wet‐aged beef (Lee et al., 2019); analysis of nonvolatile taste components of dry‐cured pork (Tian et al., 2020). John‐Lewis Zinia Zaukuu and co‐authors reported research on the optimization of an extraction technique for E‐tongue to detect beef adulterated with pork, turkey, and chicken. The results of linear discriminant analysis models on cooked meat extract was the best method for discriminating meat mixtures (Zaukuu et al., 2021). However, numerous studies have shown that deep learning algorithms are better suited for E‐tongue data analysis than simple linear models due to the high cross‐sensitivity of E‐tongue sensors (Cetó et al., 2012; Han et al., 2015; Han, Huang, Teye, Gu, Dai, et al., 2014; Han et al., 2020; Legin et al., 2004; Wesoły and Ciosek − Skibińska, 2018); Furthermore, heat‐treatment not only increase the complexity of the E‐tongue measurement but also reduces its reliability. The goal of this work was to combine E‐tongue and deep learning algorithms with the basic extraction technique, namely aqueous extraction, to detect beef adulteration. Minced beef‐meat samples were prepared and adulterated with chicken, duck, and pork separately in the range of 0 ~ 50% by weight at 10% intervals. Fisher linear discriminant analysis (Fisher LDA) and extreme learning machine (ELM) were used in comparison with modeling to distinguish between unadulterated meats and adulterated meats. When an adulterant was detected, the level of adulteration was predicted using partial least squares (PLS) and ELM and the results compared. Results show that, ELM outperformed Fisher LDA and PLS in detecting adulterated beef and predicting adulteration levels.

## 2 MATERIAL AND METHODS

### 2.1 Beef adulteration samples preparation

Silverside, pork ham, chicken, and duck legs were purchased from a local agricultural market, Suzhou, China, and transported to laboratory in an ice‐filled box. The fat and visible connective tissues of the silverside and pork ham, as well as the bones and skins of the chicken and duck legs, were removed to obtain the lean meats. Afterward, a commercial blender (JR16S‐300, Zhejiang Supor Co., Ltd., China) was used to pulverize the meats in order to prepare ground meat samples. For preparing the adulterated meats, the minced beef was mixed with chicken, duck, and pork separately in the range 0 ~ 50% by weight at 10% intervals, followed by mincing for 1 min in a blender. Twelve samples were prepared for each adulteration level of the mixed meats and the four pieces of raw meats, each with a weight of 10.0 g. This yielded a total of 228 samples for the E‐tongue measurements.

### 2.2 General chemical analysis

Total sugars, protein, fat, and ash contents were also measured in this study to validate the differences between raw meats used in basic chemical components. Total sugars were measured using the sulfuric acid‐phenol method with UV spectrophotometry in accordance with Chinese standards (GB/T 9695.31‐2008); protein, fat, and ash contents were estimated in accordance with Chinese standards 5009.5‐2016 (Kjeldahl determination), GB 5009.6‐2016 (Soxhlet extraction), and GB 5009.4‐2016 (ignition gravimetric method), respectively.

### 2.3 Taste sensors measurements and feature extraction

In this study, an E‐tongue based on multifrequency large‐amplitude pulse voltammetry (Isenso, Shanghai Ruifen International Trading Co., Ltd.) was employed. Three individual frequency segments, 1 Hz, 10 Hz and 100 Hz, were applied for the signal excitation unit. Each segment of the large‐amplitude pulse waveform had a maximal value of 1.0 V and a minimal value of −1.0 V. The taste sensors of the E‐tongue were made of metallic electrodes including platinum, gold, palladium, titanium, tungsten, and nickel, along with a reference electrode (Ag/AgCl) and a platinum counter electrode. The sensor array's cross‐sensitivity and selectivity aid in the detection of substances found in the liquid matrix, offering global chemical perception. During detection, when a voltage was applied over the working electrode and the reference electrode with the amplitude of each pulse being 0.2, a current was measured between the taste sensors and the counter electrode (Han et al., 2020; Tian et al., 2007).

Ten grams of the meat sample were blended with 100 ml of distilled water and then homogenized for 2 min using a blender (JYL‐C022E, Joyoung Co., Ltd, China). The homogenate was then centrifuged for 5 min at 10619 *g* in a centrifuge (H1850, Hunan Xiang Yi Laboratory Instrument Development Co., Ltd, China). The supernatant solution was extracted for E‐tongue measurements. Each meat sample had a detection time of 26 s (1 Hz, 23 s; 10 Hz, 2.3 s; 100 Hz, 0.23 s). For further data analysis, four points within each loop were obtained as the characteristic values of one working electrode in relation to the concentration and dispersion coefficients of the charged and electro‐active compounds in the test solution (Han et al., 2020; Tian et al., 2007).

### 2.4 Chemometrics and software

In the present work, the variable number of E‐tongue for one meat sample is 720, 40 (characteristic variables of one metal electrode) multiplied by 6 (numbers of the working electrodes), and 3 excitation frequencies (1 Hz, 10 Hz, and 100 Hz). With such high dimensional matrices as inputs, it is difficult to build a robust model for pattern recognition. Principal component analysis (PCA) was, therefore, applied to the original data firstly in order to reduce the variable numbers.

Afterward, Fisher linear discriminant analysis (Fisher LDA) and extreme learning machine (ELM) were compared for modeling to distinguish between raw and adulterated meats. When an adulterant was detected, the level of adulteration was further predicted using PLS and ELM and then compared.

Fisher LDA and PLS are the classical pattern recognition algorithms, and their foundational theory has been fully discussed in these articles (Rahim et al., 2018; Wold et al., 2001).

ELM is a popular machine learning algorithm for feed forward neural networks that combines good generalization performance with fast learning speed (Huang et al., 2011; Huang et al., 2006). The application of ELM can be divided into three parts, which are as follows. Part 1: Preparing the matrices and configuring the parameters. In the case of this study, the outputs of the E‐tongue sensors were used as input variables. The output variables were the category labels or the levels of adulteration. Other features include optimizing the number of neurons in the hidden layer and determining the hidden layer's activation function; Part 2: Network training. In this section, the input weight and bias parameters were generated at random and the connection weight between the hidden layer and the output layer was calculated to minimize the error between the network outputs and the expected ones; Part 3: Performance evaluation. For identification of the raw and adulterated meats, performances of the ELM must be evaluated by the recognition rate (%) calculated by correctly predicting the number over total number of measurements as shown in Eq. 1.

(1) $$\text{Discrimination rate}=\frac{N_{1}}{N_{2}}\times 100\%$$

where *N_1_* is the number of the correctly identified samples; *N_2_* is the number of all samples in the training set or prediction set (Han, Huang, Teye, Gu, & Gu, 2014).

For prediction of the levels of adulteration, the performance of ELM was evaluated by the root mean square error (RMSE) and the correlation coefficients (*r*) in the prediction set. The RMSE was calculated using Eq. 2,

(2) $$\text{RMSE}=\sqrt{\frac{\sum_{i=1}^{n}{\left(\overset{\wedge }{\underset{\backslash i}{y}}‐y_{i}\right)}^{2}}{n}}$$

where *n* is the number of the samples in the training set or prediction set, $y_{i}$ is the reference measurement result for the *i* ^th^ sample, and $\overset{\wedge }{\underset{i}{y}}$ is the predicted result of the model for the *i* ^th^ sample (Han, Huang, Teye, Gu, & Gu, 2014).

All algorithms in the present study were implemented in MATLAB Version 7.14 (Mathworks, Natick, USA) with windows 10.

## 3 RESULTS AND DISCUSSIONS

### 3.1 Chemicals of the raw meats

The differences in basic chemical compositions of the raw meats collected are shown in Table 1. The protein and fat contents of the raw meats differed significantly, according to the results. Results show that there were significant differences in protein and fat contents between each two. Beef and chicken had the highest protein and fat contents, while chicken and pork had the lowest protein and fat contents. The Ash content of duck and beef was found to be similar but significantly higher than that of chicken and pork. Pork had the lowest ash content. Beef and pork had the highest and lowest total sugar content, respectively. There was no significant difference in total sugar content between chicken and duck.

**TABLE 1:** Chemical analysis results of the raw meats used

	Protein (g/100 g)	Lipids (g/100 g)	Ash (g/100 g)	Total sugars (g/100 g)
Chicken	18.33 ± 0.28^a^	5.463 ± 0.09^a^	1.1 ± 0.01^a^	0.41 ± 0.02^a^
Duck	19.67 ± 0.25^b^	3.411 ± 0.06^b^	1.17 ± 0.01^b^	0.4 ± 0.01^a^
Pork	21.09 ± 0.84^c^	3.605 ± 0.1^c^	0.98 ± 0.01^c^	0.37 ± 0.01^b^
Beef	23.03 ± 0.52^d^	4.49 ± 0.11^d^	1.16 ± 0.02^b^	0.8 ± 0.01^c^

Note

Results are expressed as mean values ± standard deviation, *n* = 10. Values in the same column with different superscripts were significantly different (*p* < .05).

### 3.2 Recognition of the adulterated beef

**FIGURE 1:**
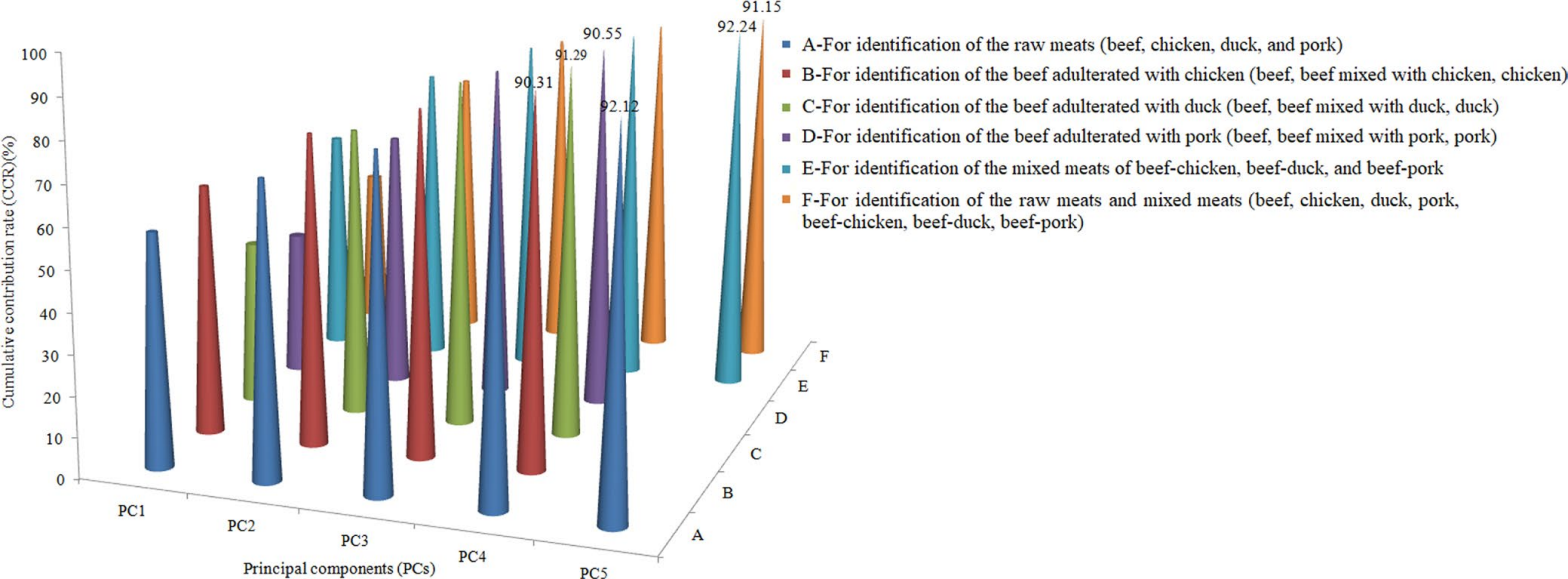
The cumulative contribution rates (CCR) of the principal components (PCs) of PCA results for different recognition targets

In this section, recognition models for raw and adulterated meats were built. PCA was primarily performed on taste sensor data matrices to reduce the dimensional structure of the input variables. The cumulative contribution rates (CCR) of the principal components (PCs) of PCA results for different recognition targets are shown in Figure 1. The top several PCs were selected for modeling, with a CCR of more than 90%. Hence, the top 4 PCs were selected to build models for identifying raw beef, beef‐meat mixtures, and raw meats, herein referred to as chicken, duck, and pork, individually; and the top 5 PCs were selected for identifying raw meats (beef, chicken, duck, and pork), mixed meats (beef‐chicken, beef‐duck, beef‐pork), and all groups of the raw meats and mixed meats. PCA results showed, the variances of the selected PCs for identification of the raw meats (beef, chicken, duck, and pork) were 57.22%, 14.81%, 8.65%, 6.83%, and 4.61%; variances of the selected PCs for identification of the beef adulterated with chicken (beef, beef mixed with chicken, chicken) were 61.84%, 14.89%, 7.72%, and 5.86%; variances of the selected PCs for identification of the beef adulterated with duck (beef, beef mixed with duck, duck) were 40.77%, 31.18%, 13.66%, and 5.69%; variances of the selected PCs for identification of the beef adulterated with pork (beef, beef mixed with pork, pork) were 36.29%, 28.03%, 19.23%, and 7.00%; variances of the selected PCs for identification of the mixed meats of beef‐chicken, beef‐duck, and beef‐pork were 56.8%, 18.81%, 9.34%, 4.71%, and 2.6%; variances of the selected PCs for identification of the raw meats and mixed meats (beef, chicken, duck, pork, beef‐chicken, beef‐duck, beef‐pork) were 40.18%, 29.39%, 12.66%, 5.62%, and 3.3%.

According to the PCA results, the top 4 or 5 PCs could explain more than 90% of the total variances of the original variables outputted by the taste sensors. This means that the E‐tongue data matrix collected is obvious collinear. Because of the inter‐sensitivity, the output of the taste electrode has a high degree of collinearity with each other. Vast amounts of redundant information not only increase the complexity of data modeling but also have impact on the prediction performance of subsequent models. These results revealed that, the six metal electrodes of the E‐tongue have nonspecific sensitivity with wide cross‐sensitivity toward the water‐soluble components in the raw meats. This also implies that each taste sensor in the array could be sensitive to the water‐soluble components extracted from the meat at the same time. On the other hand, it is possible that different taste sensors are sensitive to different water‐soluble components in the meats at the same time.

Fisher LDA and ELM were used for modeling to distinguish between raw and adulterated meats. In this work, one‐third of the samples were randomly selected as test samples, with the remaining samples as the training set for modeling. The sigmoid function as shown in Eq. 3 was utilized as the activation function of the hidden layers during ELM modeling.

(3) $$S\left(x\right)=\frac{1}{1+e^{‐x}}$$

According to ELM the theory, the number of hidden neurons has a significant impact on ELM performance. As a result, in order to achieve the best results, the number of hidden neurons was optimized by the prediction set's maximum recognition rate.

**FIGURE 2:**
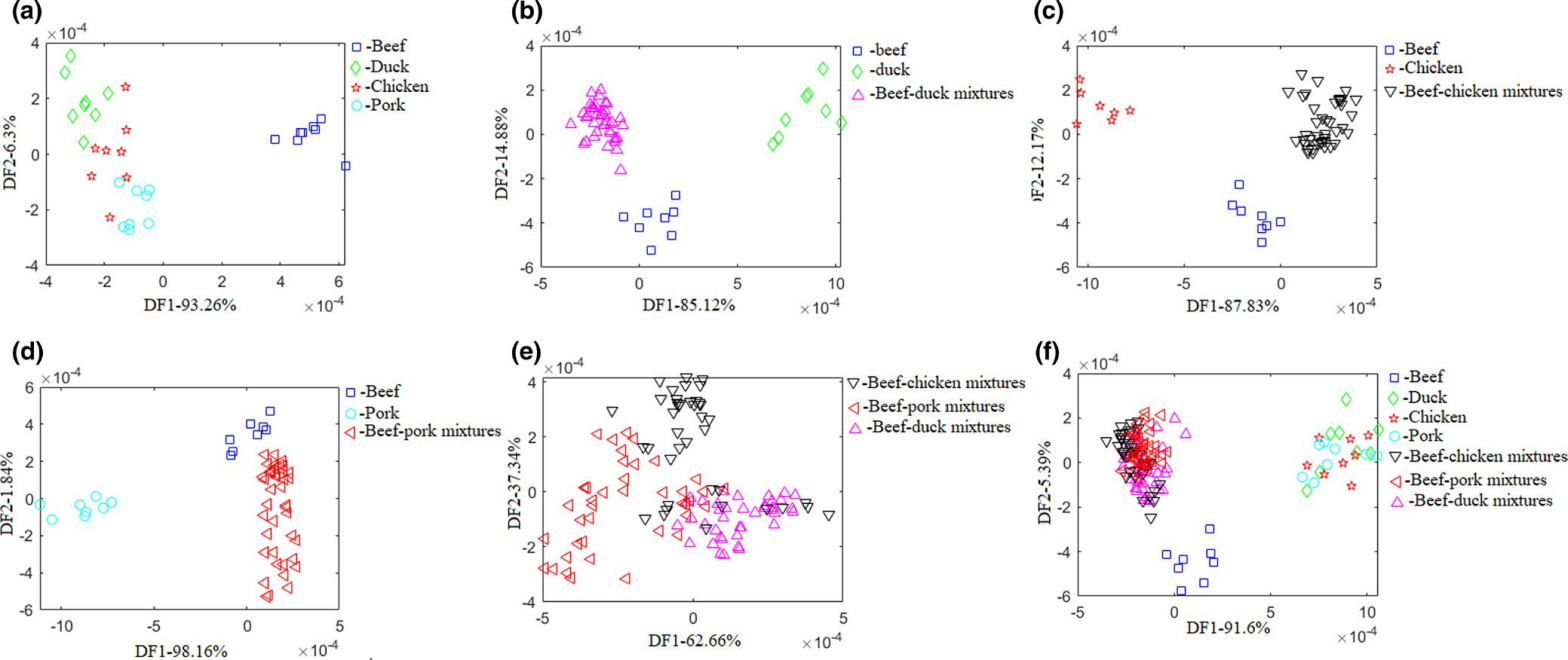
The scatter plots of the top two discriminant function (DF) scores of the E‐tongue data for various recognition targets

Figure 2 shows the scatter plots of the top two discriminant function (DF) scores of the E‐tongue data for various recognition targets. Figure 2 (B), (C), and (D) show obvious sample differentiation for the purpose of identifying raw beef, beef‐meats mixtures, and raw meats, where meat refers to chicken, duck, and pork individually. Fisher LDA results, on the other hand, were unsatisfactory for identifying raw meats (A), beef‐meats mixtures (E), and all meat groups (F).

**FIGURE 3:**
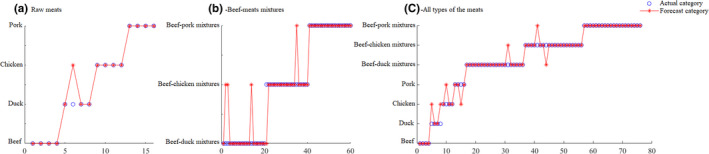
Results of the ELM models for recognition of the raw meats, beef‐meat mixtures, and all types of the meats, respectively

ELM was used to recognize raw meats, beef‐meats mixtures, and all meat groups. The results show that the optional number of hidden neurons for recognition of raw meats, beef‐meat mixtures, and all types of meats, was 16, 49, and 43, respectively. The recognition results of the test sets of the ELM models are shown in Figure 3. Table 2 shows the recognition rates of the Fisher LDA and ELM models developed for identifying raw meats and mixed meats.

**TABLE 2:** Recognition rates in the training and test sets of the Fisher LDA and ELM models built

Labels	Models	Training set (%)	Test set (%)
Beef; chicken; duck; pork	Fisher LDA	84.38 (27/32)	66.67 (8/16)
	ELM	**93.75 (30/32)**	**93.75 (15/16)**
Beef; beef‐chicken mixtures; chicken	Fisher LDA	96.43 (54/56)	96.43 (27/28)
	ELM	**100 (56/56)**	96.43 (27/28)
Beef; beef‐duck mixtures; duck	Fisher LDA	100 (56/56)	92.86 (26/28)
	ELM	100 (56/56)	**100 (28/28)**
Beef; beef‐pork mixtures; pork	Fisher LDA	89.29 (50/56)	82.14 (23/28)
	ELM	**100 (56/56)**	**96.43 (27/28)**
Beef‐chicken; beef‐duck; beef‐pork	Fisher LDA	78.33 (94/120)	65.00 (39/60)
	ELM	**95.83 (115/120)**	**91.67 (55/60)**
All types of the meats	Fisher LDA	70.40 (107/152)	69.74 (53/76)
	ELM	**92.11 (140/152)**	**90.79 (69/76)**

Note

The significance of bold values mean the superior results.

The results of the recognition models built for the identification of the four raw meats reveal that the taste sensors array utilized was suitable for measuring the difference between the water‐soluble substances extracted from the raw meats. This was mainly due to differences in the concentrations of the electrochemical compounds released from one meat to the next and/or differences in the electrochemical compounds released from each meat product, as shown in Table 1. This conclusion is based on the findings of Haddi et al. (2015) who studied the voltammograms of metal electrodes immersed in meats with species difference and quality discrepancy. Furthermore, the E‐tongue system's operating principle is a measurement of the current difference between the sensor and the reference electrode (Ag/AgCl). The interactions between chemicals in the analyzed sample and the sensor affect the taste sensor's current. Because of the current of the reference electrode is constant regardless of the sample type, the current difference measured is linked to the variation of the sensor's current, which is representative of molecules present in the analyzed sample. As a result, the voltammetric E‐tongue used in this study could be used to detect water‐soluble components in meats.

According to the recognition model results (Table 2), ELM models performed better than Fisher LDA models in the processing of E‐tongue outcomes for identification of raw meats and the adulterated beef. This is primarily due to the fact that relationships between the data matrices were more complex than linear as a result of the complexity of the test samples and the basic principles of the taste sensors, which are all partially and cross‐sensitive. ELM has a significant advantage over linear discriminant analysis algorithms for processing nonlinear problems. It indicates that the effect of prediction in the application of E‐tongue data analysis from nonlinear multivariate algorithms is better than the effect of prediction from linear multivariate algorithms due to the deep learning algorithm's superior ability of self‐learning and self‐adjusting.

### 3.3 Prediction of the adulteration levels

In this section, PLS and ELM were used in comparison with predict the adulteration levels of beef‐meat mixtures. One‐third of the samples were randomly selected as test samples, with the remaining samples serving as the training set for modeling. PLS components were optimized by cross‐validation in model calibration. The lowest RMSE value corresponds to the optimal number of PLS components. The results show that using eleven, four, and four latent variables for predicting the adulteration levels of beef‐chicken, beef‐duck, and beef‐pork, respectively, the corresponding lowest RMSE of 7.8%, 5.2%, and 7.6% could be achieved. Difference of the optimal number of PLS components between the beef‐chicken and beef‐duck or beef‐pork caused by the differences of chemical components in the binary mixture of meat samples (see Table 1). Water‐soluble proteins and vitamins, mineral salts, aliphatic acids, etc. can be easily dissolved in the distilled water. In terms of water‐soluble proteins merely, differences in charge, content, and low molecular weight protein types from four different species of meats (beef, chicken, duck, and pork) has been reported (Zhao et al., 2015). The interactions between water‐soluble compounds of the meats present in the analyzed sample and the metal electrodes affect the current of the taste sensors to output difference signals.

**FIGURE 4:**
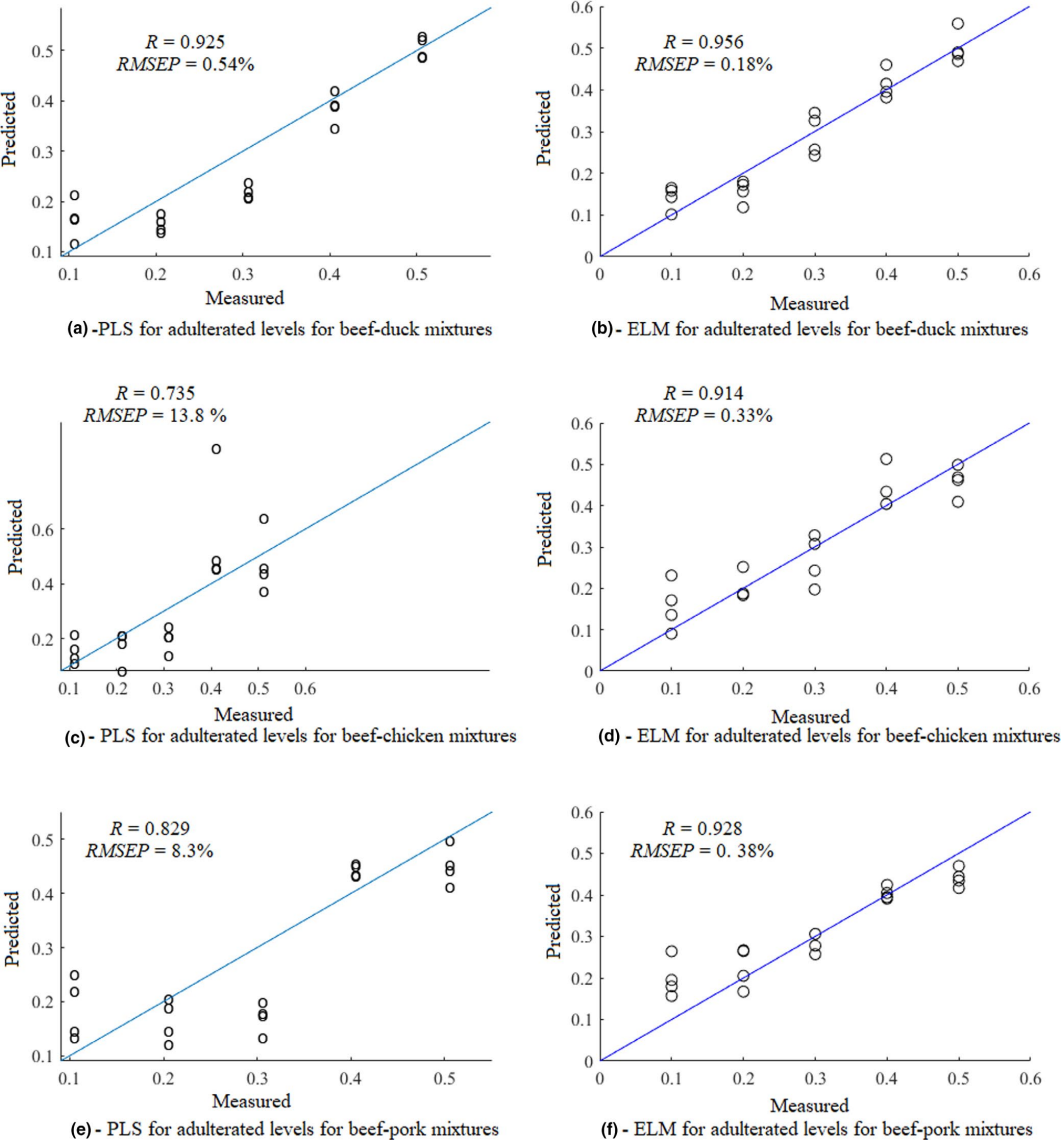
The RMSE and the correlation coefficients in the prediction set of the PLS and ELM models for prediction of the adulteration levels of the beef‐meat mixtures

The sigmoid function as shown in Eq. (4) was utilized as the activation function of the hidden layers during ELM modeling. The number of hidden neurons was determined by minimizing the prediction set's RMSE. The results show that while the number of hidden neurons was 156, 124, and 248, for prediction of the adulteration levels of beef‐chicken, beef‐duck, and beef‐pork, respectively, the optimal models could be obtained. The RMSE and correlation coefficients in the prediction set of the PLS and ELM models for predicting the adulteration levels of the beef‐meat mixtures are shown in Figure 4. It can be seen from figure that ELM models out performed PLS models in predicting adulteration levels. This is primarily due to its excellent self‐learning and self‐adjusting performance when dealing with the nonlinear issues.

## 4 CONCLUSIONS

The purpose of this study was to investigate the potential of taste sensors coupled with chemometrics for rapid determination of beef adulteration. To detect the soluble components in the meat samples studied, an E‐tongue setup based on multifrequency large‐amplitude pulse voltammetry was employed. The results show that superior recognition models based on ELM were obtained, as the recognition rates for identification of raw meats and adulterated meats were all over 90%; ELM models predicted adulteration levels of beef‐meat mixtures more precisely than PLS models, with root mean squares errors of less than 0.4 percent and coefficients of variance greater than 0.9. All of the findings indicate that taste sensors combined with ELM could be useful in the rapid detection of beef adulterated with other meats.

## CONFLICT OF INTEREST

Fangkai Han declares that there is no conflict of interest; Biao Lu declares that he has no conflict of interest; JoshuaH. Aheto declares that there is no conflict of interest; Marwan M.A. Rashed declares that there is no conflict of interest; Zhenggao Pan declares that there is no conflict of interest.

## ETHICAL APPROVAL

We the authors declare that this article does not contain any studies with human or animal subjects.

## INFORMED CONSENT

Not applicable, as this study does not include any human participants.

## Data Availability

The data that support the findings of this study are available from the corresponding author, B. L., upon reasonable request.
